# The Receptor Tyrosine Kinase RON and Its Isoforms as Therapeutic Targets in Ewing Sarcoma

**DOI:** 10.3390/cancers12040904

**Published:** 2020-04-07

**Authors:** Philipp Berning, Carolin Hennemann, Claudia Tulotta, Christiane Schaefer, Birgit Lechtape, Marc Hotfilder, Yassmine El Gourari, Heribert Jürgens, Ewa Snaar-Jagalska, Georg Hempel, Uta Dirksen, Jenny Potratz

**Affiliations:** 1 Department of Medicine A, Hematology, Oncology and Pneumology, University Hospital Münster, Albert-Schweitzer-Campus 1, 48149 Münster, Germany; 2 Department of Pediatric Hematology and Oncology, University Hospital Münster, Albert-Schweitzer-Campus 1, 48149 Münster, Germany; 3 Department of General Pediatrics, University Hospital Münster, Albert-Schweitzer-Campus 1, 48149 Münster, Germany; 4 Institute of Biology, Leiden University, Gorlaeus Laboratories, Einsteinweg 55, 2333 CC Leiden, The Netherlands; 5 Institute of Pharmaceutical and Medical Chemistry, Westfälische Wilhelms-Universität Münster, Corrensstraße 48, 48149 Münster, Germany; 6 Division of Hematology and Oncology, Department of Pediatrics III, West German Cancer Centre, University Hospital Essen, Hufelandstraße 55, 45147 Essen, Germany

**Keywords:** RON, MST1R, isoforms, IMC-RON8, narnatumab, IMC-A12, cixutumumab, Ewing sarcoma, rhabdomyosarcoma

## Abstract

The receptor tyrosine kinase (RTK) RON is linked to an aggressive metastatic phenotype of carcinomas. While gaining interest as a therapeutic target, RON remains unstudied in sarcomas. In Ewing sarcoma, we identified RON among RTKs conferring resistance to insulin-like growth factor-1 receptor (IGF1R) targeting. Therefore, we explored RON in pediatric sarcoma cell lines and an embryonic Tg(kdrl:mCherry) zebrafish model, using an shRNA-based approach. To examine RON–IGF1R crosstalk, we employed the clinical-grade monoclonal antibody IMC-RON8, alone and together with the IGF1R-antibody IMC-A12. RON silencing demonstrated functions in vitro and in vivo, particularly within micrometastatic cellular capacities. Signaling studies revealed a unidirectional IGF1-mediated cross-activation of RON. Yet, IMC-A12 failed to sensitize cells to IMC-RON8, suggesting additional mechanisms of RON activation. Here, RT-PCR revealed that childhood sarcomas express short-form *RON*, an isoform resistant to antibody-mediated targeting. Interestingly, in contrast to carcinomas, treatment with DNA methyltransferase inhibitor did not diminish but increased short-form *RON* expression. Thus, this first report supports a role for RON in the metastatic progression of Ewing sarcoma. While principal molecular functions appear transferrable between carcinomas, Ewing sarcoma and possibly more common sarcoma subtypes, RON highlights that specific regulations of cellular networks and isoforms require better understanding to successfully transfer targeting strategies.

## 1. Introduction

The receptor tyrosine kinases (RTKs) RON (also known as macrophage stimulating 1 receptor MST1R) and MET form a two-member family of so-called scatter factor receptors, based on closely related structure and function [1]. While MET signaling is a well-recognized oncogenic pathway in many cancers, first findings assigned RON a role in the regulation of innate immunity, limiting macrophage motility and inflammatory response [2,3]. Further studies demonstrated an expression in various tumors, linking RON to tumorigenesis and progression as well [4,5,6]. In epithelial cancers, RON expression has been recognized as a prognostic factor of metastasis and poor outcome [6,7]. This has been attributed to its pro-tumorigenic activities in epithelial-to-mesenchymal transition, migration, invasion and chemoresistance [4,8,9]. Targeted inhibition of RON reversed these features [8,9,10], linking RON to both progression and maintenance of an aggressive invasive-metastatic carcinoma phenotype. Moreover, recent studies involved RON in tumor–microenvironment interactions, such as angiogenesis and tumor immunogenicity, and in promoting a cancer stem cell phenotype [11,12,13]. To mediate these diverse actions, the RON receptor recruits a multitude of downstream signaling cascades. Here, central pathways such as RAS-ERK and PI3K-AKT [3,6,14] that are shared with MET and several unrelated RTKs form a basis for downstream signaling interactions. Furthermore, receptor crosstalk with RTKs, such as IGF1R [15], EGFR [16] and MET [17], has been reported, connecting RON to the broader cellular RTK network. Indeed, besides overexpression and structural variants [4,5,6], such RTK crosstalk has been implicated as a key mechanism of ligand-independent oncogenic RON activation [6,15,16,17]. Still, compared to its scatter-factor relative MET, RON and its unique ligand MSP (macrophage stimulating protein; also known as macrophage stimulating 1) [18] remain understudied and have not been investigated in sarcomas.

Ewing sarcoma is an aggressive cancer of bone and soft tissues and the second most frequent bone sarcoma in children and adolescents. Prognosis for patients with metastatic disease remains poor despite most intensive therapies [19]. Therefore, the development of IGF1R-targeted therapies sparked much excitement, given impressive responses of heavily pre-treated Ewing sarcoma patients in early clinical trials and a broad molecular basis supporting IGF1R as a tumor-driving RTK in this sarcoma [20]. However, responses in subsequent clinical trials remained below expectations [19,21]. In this context, we performed an RNA interference screen for RTKs that conferred resistance to IGF1R inhibitors in vitro and here identified RON [22]. Subsequent gene expression profiling and tissue microarray immunohistochemistry revealed RON expression in Ewing sarcoma and rhabdomyosarcoma, a second high-risk childhood sarcoma [22]. Both sarcomas share their pathognomonic molecular feature of specific chromosomal translocations that result in chimeric transcription factor oncogenes. Interestingly, both these oncogenes engage IGF1R as a cooperating signaling pathway [20]. Yet, targeted inhibition of IGF1R alone did not suffice to silence crucial downstream signaling nodes in these sarcomas in vitro. This was, however, achieved with simultaneous siRNA-silencing of RON, indicating a RON–IGF1R crosstalk with compensatory RON signaling input as an escape strategy from IGF1R inhibition [22]. Of note, in contrast to Ewing sarcoma, rhabdomyosarcomas express the RTKs EGFR and MET, the latter with specific oncogene addiction [23,24,25], placing RON–IGF1R interactions in these sarcomas before a distinct RTK network background.

Given the emerging role of RON in carcinoma progression and metastasis, but a lack of data characterizing RON in (any) sarcoma, we aimed to explore RON functions and its potential as a therapeutic target in Ewing sarcoma, alone and in its interaction with IGF1R. Indeed, our analyses demonstrate a contribution of RON to Ewing sarcoma cell migration and xenograft sarcoma burden in vivo. Yet at the same time, the clinical-grade therapeutic antibody IMC-RON8 failed to block this RON-mediated migration. Here, our study provides first evidence to hypothesize that in addition to interacting RTK networks, pediatric sarcomas express isoforms of the *RON* receptor that interfere with antibody-based targeting strategies.

## 2. Results

### 2.1. RON Expression and Activation in Ewing Sarcomas and Cell Lines

**Figure 1:**
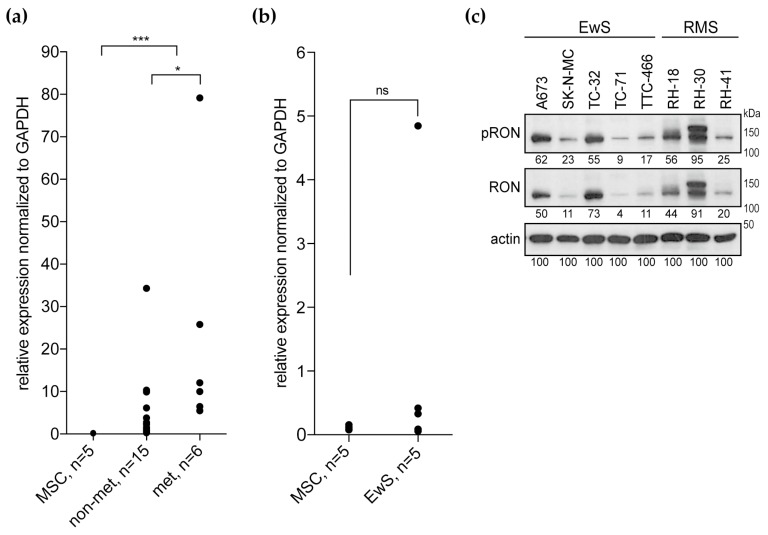
RON is expressed in Ewing sarcomas and cell lines. (**a**) Relative *RON* transcript expression in Ewing sarcoma primary tumors from patients with localized (non-met) or metastatic (met) disease in comparison to MSC cultures, as determined by qPCR. (**b**) Respective *RON* expression in Ewing sarcoma cell lines (EwS) compared to MSC cultures. (**c**) RON protein is expressed and phosphorylated in Ewing sarcoma and rhabdomyosarcoma (RMS) cell lines. Cells were grown in standard tissue culture conditions. Following analysis of phospho-RON, blots were stripped and re-probed for total RON expression; 10% gel; numbers indicate densitometry readings relative to respective actin loading control.

Our previous study found *RON* overexpressed in pediatric sarcomas compared to mesenchymal stem cells (MSCs) as a normal tissue control [22]. To confirm this microarray-based data, we analyzed *RON* transcript expression in an independent set of Ewing sarcoma tumor samples with matched clinical data using qPCR (real-time quantitative polymerase chain reaction) [23]. Relative to MSCs, *RON* was overexpressed in these tumors, and primary tumors from patients with metastatic disease showed higher *RON* levels than localized tumors (Figure 1a). A broad range of *RON* expression levels in Ewing sarcoma cell lines was not significantly different from the MSCs (Figure 1b). In two additional microarray datasets available through the R2: genomics analysis and visualization platform (https://r2.amc.nl), *RON* expression revealed broad distributions (Figure S1) [26,27]. All lines expressed the RON protein, as did the rhabdomyosarcoma cell lines, which were included into this study to address potential interaction of RON with its RTK family member MET (Figure 1c). Across this cell line panel, a relatively high-level of RON expression and activation were observed in A673, TC-32, RH-18 and Rh-30 compared to SK-N-MC, TC-71, TTC-466 and RH-41. In contrast to the *RON* transcript, *MET* expression of Ewing sarcomas and cell lines remained below the MSC levels (Figure S2). In keeping, MET protein was not detected in Ewing sarcoma cell lines, but expressed and phosphorylated in the RH-30 rhabdomyosarcoma control. Interestingly, MET was expressed but not phosphorylated in a genetically modified Ewing sarcoma cell line with shRNA-silencing of the specific Ewing sarcoma oncogene EWS-FLI1, which links the EWS-FLI1 oncogene to distinct scatter factor RTK expression patterns of pediatric sarcomas (Figure S2).

### 2.2. Functional Effects of RON Silencing

**Figure 2:**
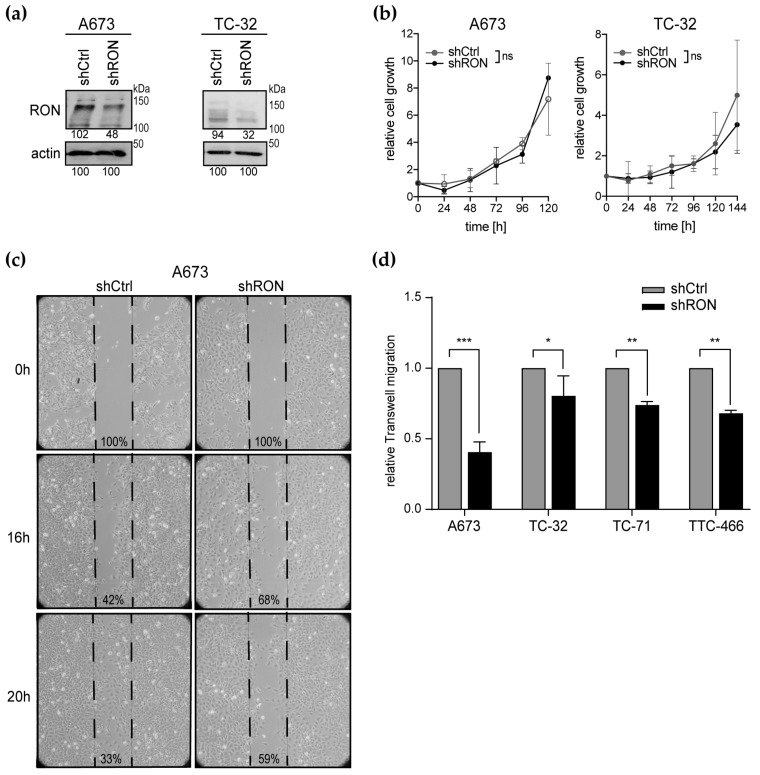
RON silencing impairs Ewing sarcoma cell migration in vitro. (**a**) RON protein knockdown 11 days after transduction with shRNAs targeting RON (shRON) or the non-silencing control (shCtrl). Numbers indicate densitometry readings relative to the respective actin loading control. (**b**) Proliferation remains unaffected by RON silencing. Cells from (**a**) were seeded into a 24-well plate at low density and one well was counted at each time point indicated (the 144 h time-point was omitted for A673 because cells were overgrown). (**c**) RON silencing delays wound healing of a confluent A673 monolayer in standard culture conditions, documented by bright-field microscopy at 40× magnification. Numbers indicate percent wound gap. Images are representative of two independent experiments. (**d**) RON silencing impairs Ewing sarcoma cell migration. Cells were cultured in serum-free medium and trans-membrane migration to serum (10%) was analyzed after 48 h. Graphs (**b**) and (**d**) represent the mean ± standard deviation (SD) of three independent shRNA transduction experiments. Significance is indicated as *p* < 0.05 (*), *p* < 0.01 (**) and *p* < 0.001 (***), while ns indicates a non-significant *p*-value.

To investigate the functional contribution of RON to Ewing sarcoma cell proliferation and metastatic capacities, we implemented RON shRNA knockdown in the A673 and TC-32 cell lines (Figure 2a). Interestingly, RON silencing did not affect monolayer cell proliferation in vitro, neither for the A673 nor TC-32 cells (Figure 2b). Addressing cellular migration as one critical step of the metastatic process, decreased RON expression delayed wound healing of the A673 cells (Figure 2c) and significantly impaired trans-well migration in four Ewing sarcoma cell lines (Figure 2d), indicating a role for RON in vitro in cellular migration rather than in monolayer proliferation.

**Figure 3:**
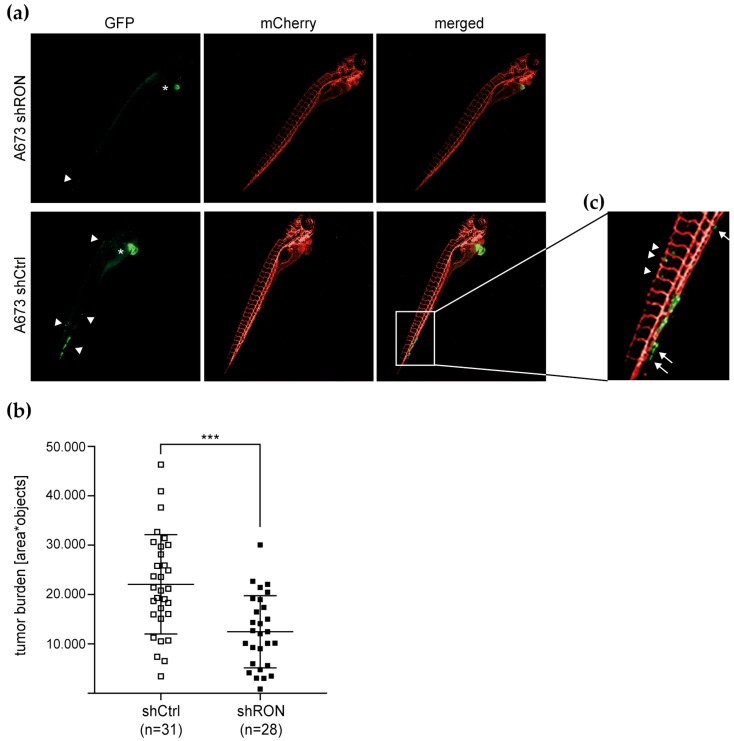
RON silencing reduces Ewing sarcoma xenograft burden in embryonic zebrafish in vivo. A673 cells expressing shRNA and concomitant GFP reporter were injected into the duct of Cuvier of transgenic zebrafish embryos with red fluorescently traced vasculature (mCherry). Zebrafish were imaged by fluorescence microscopy at 4 dpi (4× magnification): (**a**) Representative image of a xenograft-bearing zebrafish. The main tumor burden is localized in proximity to the injection site (*). Arrowheads indicate tumor cells disseminated throughout the embryo; (**b**) RON silencing reduces total tumor burden as analyzed based on GFP-fluorescent objects per zebrafish area. Bars indicate mean tumor burden ± SD of zebrafish larvae analyzed; and (**c**) representative image of Ewing sarcoma cells persisting outside the vasculature (arrowheads) and of single invasive cells no longer in contact with the endothelium (arrows).

To pursue RON’s contribution to pro-metastatic features in vivo, we chose embryonic zebrafish to model xenograft Ewing sarcoma primary tumors and micrometastases. Many cellular and molecular components of mammalian tumorigenesis and tumor suppression are conserved in zebrafish and their cancer histologies are highly similar to human cancers [28]. Following injection of tumor cells into the zebrafish blood circulation at the duct of Cuvier, the transparency of the embryonic zebrafish permits simultaneous evaluation of non-disseminated tumor cell accumulations close to the injection site that correspond to localized tumor burden and of tumor cells disseminated throughout the embryo that represent (micro-) metastases [29]. At the same time, genetically engineered zebrafish lines with tissue-specific reporters enable high-resolution in vivo analysis of tumor cell interactions with the host microenvironment during tumor progression [28,30]. Employing a previously established model [29,31], we injected A673 cells expressing shRNA silencing RON or non-silencing control together with green-fluorescent reporter protein (GFP) into a transgenic zebrafish line with red fluorescently traced endothelium (Figure 3). Analysis of total tumor burden was performed at 4 days post implantation (dpi) on whole embryos (Figure 3a) and revealed that reduced RON expression significantly reduced total tumor burden (Figure 3b). As previously described [29], disseminated tumor cells were found predominantly at the posterior ventral end of the caudal hematopoietic tissue (CHT) in the zebrafish tail (Figure 3a,c). Interestingly, extravasation of implanted cells from the host vasculature has been reported as a phenomenon independent of cellular tumorigenic properties. However, such cells disappeared before 4 dpi and only perivascular cells with moderate or high metastatic potential were capable of invading the neighboring tail fin tissue to subsequently develop micrometastasis [29,32]. While we did not observe formation of multi-cellular micrometastases by our time point of analysis at 4 dpi, CHT areas showed A673 cells harboring non-silencing shRNA that persisted outside the vasculature (Figure 3c). Moreover, single invasive cells that had lost contact with the endothelium and entered into the tail fin tissue were observed (Figure 3c). Although these dissemination and invasion capacities could not be conclusively quantified, they appeared reduced with RON silencing, suggesting that RON contributes to tumor burden in vivo and to the micrometastatic potential of Ewing sarcoma cells.

### 2.3. Activity of the Therapeutic Antibody IMC-RON8 In Vitro

**Figure 4:**
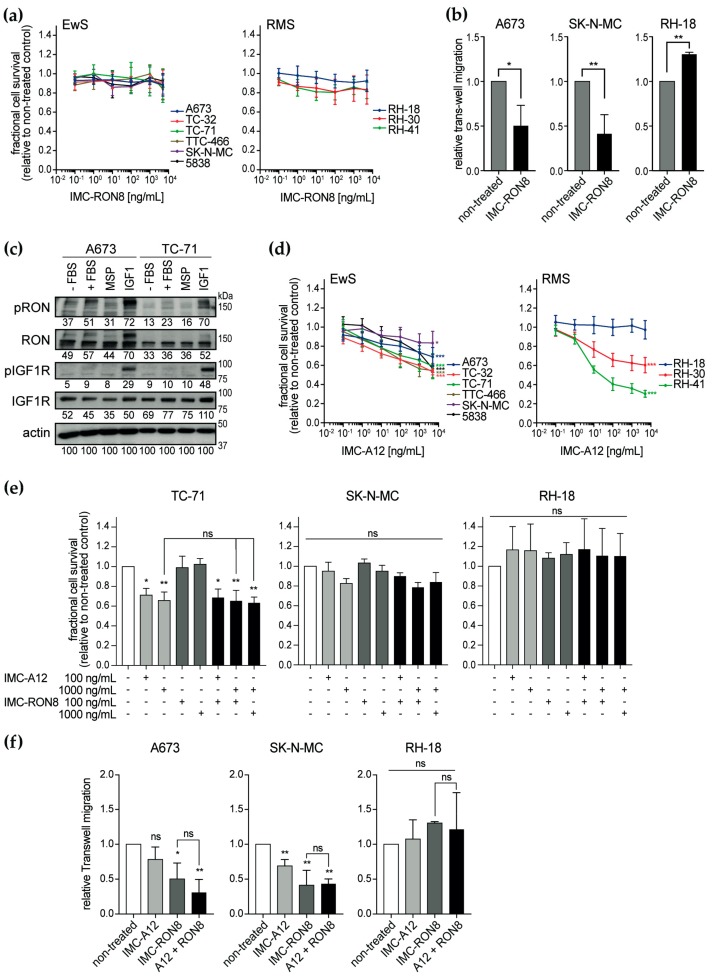
IMC-RON8 antibody inhibits migration but not proliferation of Ewing sarcoma cell lines in vitro. (**a**) IMC-RON8 does not significantly reduce monolayer cell viability of EwS and RMS cell lines. Cells were grown in standard conditions and treated as indicated. After 72 h, relative cell viability was measured by MTT assay. (**b**) IMC-RON8 impairs migration of Ewing sarcoma cells. Cells were cultured in serum-free medium, seeded into trans-well chambers containing 100 ng/mL IMC-RON8 where indicated and allowed to migrate towards 10% serum. Graphs represent mean ± SD of triplicate experiments. (**c**) RON is cross-activated by IGF1–IGF1R signaling but not vice versa. Cells were starved in serum-free medium for 24 h before stimulation with fetal bovine serum (FBS) (10%), MSP (400 ng/mL) or IGF1 (100 ng/mL) for 30 min. Following analysis of phospho-RON, blots were stripped and re-probed for total RON expression; numbers indicate densitometry readings relative to respective actin loading control. (**d**) Dose-response of the EwS and RMS cell lines to the anti-IGF1R antibody IMC-A12. Assays were performed as in (**a**); significances refer to maximum dose compared to non-treated cells. (**e**) Combined treatment with IMC-RON8 plus IMC-A12 does not result in synergistic effects on cell viability. MTT assay was performed as in (**a**). Graphs (**a**), (**d**) and (**e**) represent the mean ± SD of at least three independent experiments. (**f**) Combined treatment with IMC-RON8 plus IMC-A12 does not reveal synergistic effects on migration. Assays were performed as in (**b**) with 100 ng/mL IMC-A12 and 1000 ng/mL IMC-RON8 as indicated. Significance is indicated as *p* < 0.05 (*), *p* < 0.01 (**) and *p* < 0.001 (***), while ns indicates a non-significant *p*-value.

To explore RON as a therapeutic target, we utilized the monoclonal antibody IMC-RON8 (narnatumab) that had reached phase I clinical investigation [33]. IMC-RON8 blocks MSP–RON binding and thus ligand-induced receptor activation and signaling. However, IMC-RON8 did not significantly affect monolayer cell viability in vitro in any of the nine pediatric sarcoma cell lines analyzed (Figure 4a), independent of their baseline RON expression and activation (Figure 1c). Colon (HT-29, HCT-116) and pancreatic (CAPAN-1) adenocarcinoma cell lines, examined as controls with well-characterized oncogenic RON function, remained similarly unaffected (Figure S3). Yet, concordantly with shRNA-based findings, IMC-RON8 significantly impaired migration of Ewing sarcoma cell lines (Figure 4b), while interestingly promoting migration of RH-18 rhabdomyosarcoma cells.

Because our previous study had shown that RON provided compensatory signaling for loss of IGF1R input [22], we speculated whether here in turn IGF1R signaling attenuated IMC-RON8 efficacy and whether this may then be restored through simultaneous targeting of IGF1R. Evaluating both RTKs for co-activations (Figure 4c), RON showed little constitutive activation in the absence of serum and a surprisingly weak response to stimulation with its specific ligand MSP. Somewhat more prominent activation was observed with serum stimulation, whereas strongest activation occurred following IGF1 treatment. In contrast, substantial IGF1R activation was restricted to its specific ligand. These findings indicate a unidirectional cross-activation of RON by IGF1–IGF1R signaling. 

To target this cross-activation, we employed the monoclonal antibody IMC-A12 (cixutumumab) [34], which had reached phase II clinical studies. In a mechanism of action similar to IMC-RON8, IMC-A12 blocks IGF1 ligand–IGF1R receptor binding and signaling activation. Evaluating the effect of IMC-A12 on cell viability (Figure 4d), Ewing sarcoma cell lines revealed an overall intermediate response. Here, the TC-32 and TTC-466 (half-maximal inhibitory concentrations (IC_50_) < 10 µg/mL), 5838 and TC-71 (IC_50_ < 20 µg/mL) cell lines were more sensitive compared to more resistant A673 and SK-N-MC cells (IC_50_ > 100 µg/mL). Rhabdomyosarcoma cell lines presented a broader spectrum of sensitive (RH-41; IC_50_ < 0.1 µg/mL), intermediate (RH-30; IC_50_ < 20 µg/mL) and resistant lines (RH-18; IC_50_ undetermined). However in contrast to our hypothesis based on prior co-targeting of RON with siRNA and IGF1R tyrosine kinase inhibitor [22], combined treatment with IMC-RON8 and IMC-A12 did not overcome resistance or enhance effects on cell viability compared to single antibodies (Figure 4e). Similarly, parallel shRNA-silencing of RON did not alter the IMC-A12 effects on cell proliferation in vitro (Figure S3). Regarding cell migration, IMC-A12 alone had a significant inhibitory effect only on SK-N-MC, and thus not on A673 or RH-18 cells. Again, antibody-mediated co-targeting of both RTKs did not reveal synthetic inhibition of cell migration compared to RON targeting alone (Figure 4f).

Given that MET was not active or expressed at substantial levels in Ewing sarcoma (Figure S2), we had included MET-driven rhabdomyosarcoma cell lines into this study [25], hypothesizing that rhabdomyosarcomas would utilize compensatory MET signaling and therefore be less amenable to RON and IGF1R co-targeting than Ewing sarcoma. However, with the significant effects of IGF1R co-targeting not observed in MET-negative Ewing sarcoma counterparts, this setting appeared unsuitable to pursue potential RON−IGF1R−MET interaction in rhabdomyosarcoma.

Taken together, IMC-RON8 studies recapitulate shRNA-based findings in indicating a role for RON in the migratory phenotype of Ewing sarcoma. Yet, although our data reveal RON activation through both MSP and IGF1 ligand-mediated mechanisms, IMC-A12 failed to sensitize sarcoma cells to IMC-RON8. While this may be due to different strategies of IGF1R inhibition (ligand-dependent antibody versus tyrosine kinase inhibitor in the prior study), our current focus on RON led us to question whether additional ligand-independent mechanisms contributed to net RON activity and attenuated the efficacy of both antibodies.

### 2.4. Expression of RON Isoform Variants in Ewing Sarcoma

**Figure 5:**
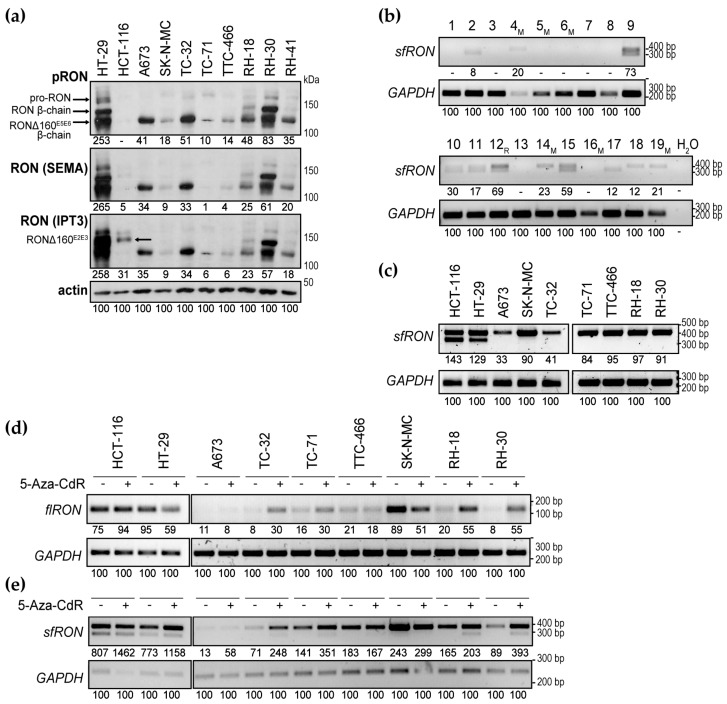
Ewing sarcomas express targeting-relevant RON isoforms. (**a**) Western blots suggest the presence of full-length RON (flRON) splice variants in pediatric sarcoma cell lines. RON protein expression was analyzed in comparison to characterized isoforms in HT-29 and HCT-116. Cells were grown in standard tissue culture conditions. Following analysis of phospho-RON, Western blots were stripped and re-probed for analysis of two distinct total RON antibodies directed at the SEMA and IPT3 domain epitopes. Arrows indicate RON species. 8% gel. (**b**) Ewing sarcomas express the short-form *RON* (*sfRON*) isoform containing (upper band) and/or lacking intron 11 sequences (lower band). Tumor samples are numbered; M indicates primary tumors from patients with metastatic disease, R indicates a relapsed tumor. (**c**) Sarcoma cell lines express *sfRON*. RT-PCR was performed on mRNA isolated from cell lines grown in standard conditions. (**d**,**e**) Treatment with 5-Aza-2’-deoxycytidine (5-Aza-CdR) modulates *flRON* (**d**) and *sfRON* (**e**) transcription. RT-PCRs were performed on mRNA isolated from cell lines grown in standard conditions and treated with 2.5 µM 5-Aza-CdR for 72 h where indicated. In (**a**–**e**), numbers indicate densitometry readings relative to the respective actin or *GAPDH* loading control.

RON isoform variants with ligand-independent activities bear the potential to subvert the effects of IMC-RON8 or other compounds that target ligand–receptor binding. In carcinomas, at least eight isoforms have been reported, most due to alternative splicing. Several exhibit constitutive tyrosine kinase activity or confer resistance due to intracellular localization or loss of N-terminal ligand-binding elements [6,35,36,37]. As a first step to address such isoforms in pediatric sarcomas, we re-examined whole cell protein lysates of cell lines at a higher separation and in comparison to characterized RON isoforms (Figure 5a): HT-29 cells express the 180 kDa (kilodalton) wild-type pro-RON precursor, which is cleaved into the 40 kDa α-chain and the 150 kDa wild-type RON β-chain [18]. At the same time, HT-29 cells co-express the Δ160^E5E6^ variant. Alternative splicing with deletion of exons 5-6 (corresponding to the IPT1 domain) results in a 160 kDa precursor chain that is cleaved into the α-chain and a constitutively active 125 kDa β-chain [35]. HCT-116 cells are characterized by the splicing variant Δ160^E2E3^ with deletion of exons 2–3 (corresponding to parts of the β-chain SEMA domain). It forms an un-cleaved single chain protein of 160 kDa that is retained in the intracellular compartment, shows no tyrosine phosphorylation ability, but is associated with constitutive AKT activation [36]. In keeping, Western blots of Figure 5a revealed the Δ160^E5E6^ variant with phospho-specific and both SEMA and IPT3 domain-directed antibodies, whereas the Δ160^E2E3^ variant was better visualized with an IPT3 epitope-directed antibody. Interestingly, all Ewing sarcoma and RH-41 rhabdomyosarcoma cells expressed a single phosphorylated protein band of similar weight as the 125 kDa Δ160^E5E6^ β-chain, while RH-18 and RH-30 showed a second band corresponding in weight to the 150 kDa β-chain of wild-type RON. Although these preliminary findings require further validation of specific isoforms, they suggest that pediatric sarcomas may express splicing variants of full-length RON (flRON) that evade targeting strategies based on interruption of ligand–receptor binding, such as IMC-RON8.

Short-form RON (sfRON) is a distinct truncated isoform that derives not from alternative splicing but an alternative intragenic promoter between introns 8 and 10 [5,38]. Functionally, sfRON is of particular interest due to its strong constitutive tyrosine kinase activity that was shown to confer an aggressive, motile phenotype in several carcinomas [5]. Moreover, it lacks all extracellular domains, so that the sfRON proportion of total RON activities may undermine therapeutic benefits derived from flRON-directed N-terminus-targeting strategies, such as IMC-RON8. We therefore investigated *sfRON* expression in Ewing sarcomas and found *sfRON* expressed in 11 of 19 Ewing sarcoma tumor samples (Figure 5b). Sequencing of the double bands revealed *sfRON* variants with and without splicing of intron 11 (Figure S4), as previously reported in carcinomas [5]. Cell lines of Ewing and rhabdomyosarcoma expressed *sfRON* as well (Figure 5c). Interestingly, in contrast to tumor samples and to HT-29 and HCT-116, they displayed a single-banded pattern of intron 11-containing *sfRON* only.

### 2.5. Modulation of RON Isoform Transcription by 5-Aza-CdR

In carcinomas and leukemias, differential expression of full-length *RON* (*flRON*) and *sfRON* isoforms has been found governed, at least in part, by methylation patterns of two CpG islands in the proximal *RON* promoter. Hypermethylation of island 1 coincided with lack of *flRON*, whereas hypermethylation of island 2 was associated with *sfRON* transcription [38]. Hence, modulation of DNA methylation was explored as a means to shift *RON* isoform patterns. In erythroleukemic cells expressing *sfRON* but not *flRON*, treatment with the DNA methyltransferase inhibitor 5-Aza-2’-deoxycytidine (5-Aza-CdR; decitabine) inverted isoform expressions, downregulating *sfRON* and upregulating *flRON* [38]. Yet conversely, treatment of initially sfRON-negative myeloid leukemia cells induced sfRON expression [39]. We therefore tested whether 5-Aza-CdR affected isoforms of pediatric sarcoma cell lines in favor of the IMC-RON8-sensitive *flRON*. For *flRON* analysis, we here used a primer pair that anneals in exon 20 and remains unaffected by the alternative-splicing variants of *flRON* discussed above. Expression levels *flRON* appeared lower in sarcoma compared to carcinoma cells and indeed increased following 5-Aza-CdR treatment in several cell lines (Figure 5d), whereas relatively higher-level *flRON* expression of HT-29 and SK-N-MC decreased (or remained unchanged). Interestingly, because contrary to published data from leukemic cell lines, treatment with 5-Aza-CdR did not diminish *sfRON* transcription, but instead upregulated *sfRON* in most sarcoma cell lines. Furthermore, 5-Aza-CdR induced splicing of intron 11 in several sarcoma cell lines (lower-base pair *sfRON* band; Figure 5e). These data indicate that similar to carcinomas and leukemias, expression of *RON* species in sarcomas underlies, at least in part, transcriptional regulation by methylation. Yet in contrast, 5-Aza-CdR treatment failed to diminish intrinsically IMC-RON8-resistant *sfRON*.

## 3. Discussion

### 3.1. RON as a Therapeutic Target in Ewing Sarcoma Metastasis

We report the first analysis of RON expression and function in sarcomas, specifically Ewing sarcoma. Notably, although *RON* was overexpressed compared to MSC in several tumor samples (particularly from metastatic disease), this does not constitute genuine overexpression, as the relationship between Ewing sarcoma and normal bone marrow-derived MSC remains unclear. Although *RON* overexpression is established for various cancers [3], a query of the cBioPortal platform [40,41,42] revealed that overexpression due to genomic amplification is generally rare and was it not observed in two large-scale datasets of 219 Ewing sarcomas and cell lines accessible through the platform [43,44]. Furthermore, the MSK-IMPACT Clinical Sequencing Cohort of >10,000 cancer patients available at cBioPortal revealed a low *RON* mutation frequency of <2% that did not influence survival. Interestingly, no more than two mutations were found in Ewing sarcoma datasets and none in established cell lines. Thus, RON lacks oncogenic amplification or mutation to advocate it as a classical tumor-driving molecular target in Ewing sarcoma. Furthermore, RON silencing (shRNA- or antibody-mediated) did not affect cell viability or proliferation of monolayer cultures in vitro. Yet in our in vivo model, RON silencing reduced tumor burden. Importantly, this tumor burden does not reflect proliferation alone but provides a combined measure of xenograft tumor−host interaction, cell survival, proliferation and death. The above findings are in keeping with pancreatic carcinoma, where RON is a well-characterized molecular target. Although neither IMC-RON8 nor RNAi knockdown affected monolayer cell proliferation in vitro [45], RON silencing inhibited tumor growth of pancreatic cancer mouse xenografts in vivo, primarily due to an increased susceptibility to apoptosis [9]. Furthermore similar to its ascribed role in carcinomas, our in vitro and in vivo data, despite important limitations as to a single cell line, animal number and quantification of distant-site invasion, indicate that RON contributes to the micrometastatic properties of Ewing sarcoma cells, illustrating its scatter factor nature [6]. Following its characterization in diverse carcinomas and leukemia, our study therefore recommends further investigation of RON as a molecular component in the progression of sarcomas, possibly with a more prominent role in metastasis than in proliferation of established tumors or as an oncogenic driver-RTK. 

Following the starting point presented here, subsequent comprehensive analyses of RON function in pediatric sarcomas should include alternative shRNA sequences or transgenic rescue control and the design of such future RNA interference or CRISPR sequences should account for the predominant isoforms at play. In vitro, anchorage-independent spheroid or soft-agar colony assays that explore suppression of anoikis and tumorigenicity as additional prerequisites of metastasis will be useful. In vivo, our experiments in embryonic zebrafish, despite their limitations, underline the principal value of this model for the study of micrometastatic sarcoma progression. However, to study specific mechanisms responsible for decreased tumor burden, to quantify and analyze xenograft metastases tissue (e.g., for specific isoform expressions) and to explore the anti-tumor activity of alternative RON-targeting strategies, mouse xenograft models will be more suitable.

### 3.2. Targeting RON with the IMC-RON8 Antibody Strategy

Activity of the therapeutic IMC-RON8 antibody in Ewing sarcoma cell lines correlated with shRNA-based experiments, inhibiting their migration but not proliferation. This is in line with a previous report in pancreatic cancers cells, where IMC-RON8 treatment in vitro impaired MSP-induced RON signaling and migration but not proliferation [45]. Yet while both cancers’ migratory phenotypes showed some response to IMC-RON8, simultaneous MSP-independent RON activities may undermine IMC-RON8’s net inhibitory effect. Although requiring further validation, our findings suggest that analogical to pancreatic and other carcinomas [5,6,17], RON isoforms and RON–RTK networking are general principles of such MSP-independent RON activation in sarcomas. To successfully pursue IMC-RON8’s ligand-directed targeting strategy, it will therefore be crucial to define confounding RTKs and RON isoforms or certain patterns thereof that perform as biomarkers of resistance or response. Yet, of note, clinical development of IMC-RON8, the only RON-targeting monoclonal antibody to reach clinical trial, has been discontinued [46]. Indeed, given the kinase-mediated nature of resistance mechanisms, a kinase-directed targeting strategy in turn may be of advantage. Chakedis et al. recently reported promising results with a small-molecule tyrosine kinase inhibitor that inhibited MSP ligand-mediated RON kinase activation as well as the constitutive kinase activity of RON isoforms, including sfRON, in pancreatic cancer cell lines [37]. Due to the structural similarity of the tyrosine kinase domains across RTK families, most tyrosine kinase inhibitors lack specificity compared to monoclonal antibodies. While therefore being less suitable when studying specific RON functions, a broader target spectrum comprising RON among other, more-established RTK targets (including its interacting co-targets MET and IGF1R [6,47]) may promote the clinical development of tyrosine kinase inhibitors, such as crizotinib (NCT02612194) or ASLAN002 (also known as BMS-777607; NCT01721148) [48].

### 3.3. RON Acts as One Member of a Cellular RTK Network

We show that RON connects to the RTK network of Ewing sarcoma cells to gain MSP-independent activation. One, though likely not exclusive, interacting partner is IGF1R, a prominent RTK in Ewing sarcoma biology [20]. Our previous study had shown that therapeutic inhibition of IGF1R kinase activity resulted in compensatory RON input to shared downstream signaling nodes, such as RPS6 [22]. This current study now indicates a second, more upstream receptor-level interaction through IGF1−IGF1R signaling-mediated cross-activation. This mechanism was unidirectional and has recently been first reported in pancreatic cancer cells, where RON was found to serve as a mediator of IGF1R signaling [15]. Yet despite strong cross-activation from IGF1−IGF1R signaling, IMC-A12 was unable to sensitize sarcoma cells to RON targeting and showed limited effects as a single agent. This is in contrast to our previous study, where an IGF1R tyrosine kinase inhibitor markedly sensitized cells to RON knockdown [22]. Based on our limited analysis so far and on the literature from other cancers [49,50], one explanatory hypothesis is that both RON and IGF1R act as members of a redundant signaling network that maintains and compensates signaling input across its cascades. Because IMC-A12 and IMC-RON8 are by mechanism of action restricted to ligand-mediated signals, cross-activation between tyrosine kinases may pass by, leaving the sensitizing capacity of the antibodies as inferior to the kinase inhibitor approach of our prior study. To answer this, a systemic analysis of RON signaling activation (serum, specific ligands, starvation and RTK cross-talk), transduction (through common signaling nodes down to effectors such RPS6 [22]) and response (to ligand-dependent antibodies versus specific or broad-spectrum tyrosine kinase inhibitors) is warranted, but exceeded our study.

### 3.4. Targeting-Relevant RON Isoforms in Ewing Sarcoma

This study provides, to our knowledge, first evidence of targeting-relevant RTK isoforms in pediatric sarcomas. Although the migratory phenotype of Ewing sarcoma was sensitive to IMC-RON8, indicating ligand-dependency at least in part, simultaneous expression of constitutively active isoforms may dampen the efficacy of ligand-directed targeting strategies. To clearly define wild-type RON and individual flRON splicing variants suspected in Ewing sarcoma cell lines, despite overlapping molecular weights and lack of specific antibodies, isoform-specific PCRs and more advanced proteomic techniques will be required. This will be important, as most flRON splice variants confer ligand-independent, constitutive tyrosine kinase activity [6]. Short-form RON, unsusceptible to immunoblot detection using N-terminus-directed antibodies as chosen for this study, remains to be confirmed at the protein level. Therefore, our data so far provide no more than starting-points for future analyses: To distinguish the exact functional significance of specific isoform variants on the proliferative, migratory or metastatic phenotype of Ewing sarcoma, signaling crosstalk, drug response, selective depletion or overexpression experiments are warranted. To assess a clinical relevance, isoform analyses in tumor tissue with corresponding clinical data will be important. In these analyses, sfRON deserves particular interest for conferring resistance to N-terminus-directed therapeutic strategies [5,6].

Modulating *RON* promoter methylation to shift relative *sfRON* and *flRON* expressions towards a more drug-sensitive pattern provides an intriguing strategy [37,38,39]. Yet, as previously noted in pancreatic cancer, cell line-specific responses are observed [37]. In most Ewing sarcoma cell lines, DNA methyltransferase inhibition did not prompt a shift between isoforms, but an increase in both *sfRON* and *flRON*. In principle, this up-regulation of *RON* species as therapeutic targets may sensitize sarcoma cells to tyrosine kinase inhibitors that silence both MSP-mediated and MSP-independent tyrosine kinase activities (in contrast to the IMC-RON8 antibody). Indeed, in myeloid leukemia cells, 5-Aza-CdR has been used to produce sfRON as de novo target for tyrosine kinase inhibitor treatment [39]. However, a potential benefit of this approach must be carefully weighed against the risk of enhancing oncogenic RON actions. As yet, confirmatory analyses of actual RON promoter methylation in sarcomas, protein expression and signaling outcome of DNA methyltransferase inhibition remain open. Particularly, the functional consequences of *sfRON* (and *flRON*) induction on the metastatic ability of Ewing sarcoma require investigation. Furthermore, in light of an increasing therapeutic exploitation of DNA (de-) methylation, global effects on tumor phenotypes, beyond RON and RTKs, require consideration.

The sfRON isoform has been shown to interact with the cellular tyrosine kinase network. In gastric cancer, specifically sfRON was found to confer resistance to therapeutic MET targeting [51]. While flRON acted transactivated by MET and MSP ligand failed to reactivate flRON in the presence of a MET inhibitor, sfRON maintained downstream signaling and conferred resistance. In myeloid leukemia, sfRON but not flRON physically interacted with the intracellular tyrosine kinase LYN to drive cell proliferation in a PI3K/AKT-independent manner [39]. Before, interactions with IGF1R, EGFR and MET receptors had been shown for flRON [15,16,17] and several studies had demonstrated MSP-mediated tumor proliferation and progression involving the PI3K/AKT signaling pathway [6,10]. Thus, MSP-activated RON and sfRON may act as original tyrosine kinases with distinct signaling and interaction partners. To take this further, Angeloni et al. reported that RON species affected each other, with flRON reducing protein expression and kinase activity of its sfRON counterpart [38]. Thus, in light of increasingly emerging data on sfRON as an intriguing new target and important mechanism of resistance, it will be important to unravel which cellular functions and network interactions of RON are truly MSP ligand-mediated, which are shared between flRON and sfRON, or unique to each isoform.

## 4. Materials and Methods

### 4.1. Cell Lines and Tumor Samples

Ewing sarcoma and rhabdomyosarcoma cell lines were originally received from the cell culture bank at Children’s Hospital Los Angeles (see DSMZ and ATCC for reference). HT-29 and HCT-116 colon carcinoma cell lines were from the Leibniz Institute DSMZ-German Collection of Microorganisms and Cell Cultures (DSMZ, Braunschweig, Germany) and CAPAN-1 pancreas adenocarcinoma cells were from American Type Culture Collection (ATCC, Manassas, VA, USA). Short tandem repeat profiling was performed to verify identities and cells were regularly tested to be free of mycoplasma. Standard cell culture conditions were in RPMI1640 medium with 10% fetal bovine serum (FBS) (Invitrogen, Carlsbad, CA, USA) at 37 °C and with 5% CO_2_. Experiments we performed at ~80% cell confluence. Ewing sarcoma tumor samples, MSC cultures and respective ethical approval were previously described [23].

### 4.2. Compounds and Reagents

IMC-RON8 [33] and IMC-A12 [34] monoclonal antibodies were provided by ImClone Systems Corporation (Branchburg, NJ, USA) through a Material Transfer Agreement. Recombinant human MSP (Cat-No. 352-MS) was from R&D Systems (Minneapolis, MN, USA) and recombinant human insulin-like growth factor 1 (IGF1; Cat-No. I3769) and 5-Aza-2’-deoxycytidine (5-Aza-CdR, Decitabine; Cat-No. A3656) were from Sigma-Aldrich (St. Louis, MO, USA).

### 4.3. shRNA Plasmids and Lentiviral Transduction

GIPZ^TM^ lentiviral shRNA plasmids and packaging plasmids (Trans-Lentiviral Packaging Kit, Cat-No. TLP5912) were from GE Healthcare Dharmacon (Lafayette, CO, USA). GIPZ^TM^ plasmids express the turbo GFP reporter protein as part of a bicistronic transcript with shRNAs. RON was targeted using a pool of three distinct shRNAs (V3LHS_643802; V3LHS_646375; V3LHS_643804). A non-silencing shRNA (Cat-No. RHS4346) served as control. shRNA plasmids were purchased as glycerol stocks, prepared according to the manufacturer’s protocol, and inserts were sequence-confirmed prior to use. Generation of lentivirus and spin transductions were performed as previously described [52], with the modification that after centrifugation, plates were incubated at 4 °C for 30 min before incubation at 37 °C with 5% CO_2_ overnight. A second transduction was performed on Day 1. Experiments were performed as of Day 9.

### 4.4. Reverse Transcription PCR and Sequencing

RNA isolation, Moloney murine leukemia virus (M-MLV) reverse transcription, qPCR, RON, MET and GAPDH primers, probes spanning exon-exon junctions and analysis relative to GAPDH housekeeping gene were performed as recently reported [23]. Reverse transcription PCR (RT-PCR) of short-form RON (sfRON) was carried out as semi-nested PCR as described by Bardella et al. [5]. Each 25 µL PCR reaction contained 2 µL template, 200 µM dNTP, 0.2 µM primers, 1.5 mM MgCl2 and 0.6 units of GoTaq^®^ G2 Flexi DNA polymerase in 1× buffer (Promega, Madison, WI, USA). Product cleanup used the GeneJET PCR Purification Kit (Thermo Fisher Scientific, Waltham, MA, USA) according to the manufacturer’s protocol. To sequence *sfRON* PCR products, bands were excised from agarose gels and PCR products were extracted using the QIAquick Gel Extraction Kit (Qiagen, Hilden, Germany). Sequencing was performed in both directions using sfRON sense and exon 12 antisense primers as previously described [52]. Sequences were analyzed using CodonCode Aligner v8.0.2. For comparative RT-PCR of full-length RON, we chose primers annealing on a C-terminal exon 20 sequence: 5’-TAGTGTCTGCACTGCTTGGG (forward) and 5’-GCTGTTCTGGACGCACATTC (reverse) [37].

### 4.5. Western Blotting

Procedures and buffers were as previously described [22]. Primary antibodies detecting RON were Cat-No. HPA007657 (SEMA domain amino acids 283-433, corresponding to exons 1-2; unless otherwise specified, this antibody was used for total RON detection) and Cat-No. HPA008180 (IPT3 domain amino acids 767–875, corresponding to exons 9–10) from Sigma-Aldrich; phospho-RON (kinase domain Tyr1238/39) (Cat-No. AF1947) was from R&D Systems; MET (25H2) (Cat-No. 3127), phospho-MET (Tyr1234/35; D26) (Cat-No. 3077) and phospho-IGF1R (Tyr1131/1146) (Cat-No. 3021) were from Cell Signaling Technology (Beverly, MA, USA); IGF1Rβ (C20) (Cat-No. sc-713) and actin (C4) (Cat-No. sc-47778) were from Santa Cruz Biotechnology (Santa Cruz, CA). Secondary horseradish-peroxidase-conjugated antibodies were anti-mouse (Cat-No. 7076, Cell Signaling Technology) and anti-rabbit (Cat-No. 554021, BD Pharmingen, Franklin Lakes, NJ, USA). Densitometric analyses were performed using Image J software (version 1.52j). Uncropped blots are provided in Figure S5.

### 4.6. Cell Viability Assay

Cells were seeded into 96-well plates, at densities of 2–5 × 10^3^ cells per well in 100 µL of standard growth medium containing 10% FBS. Cells were allowed to attach for 24 h before treatment. After 72 h of treatment, cell viability was determined by a standard 3-(4,5-Dimethylthiazol-2-yl)-2,5diphenyltetrazolium-bromid (MTT; Sigma-Aldrich) assay. Absorbance was measured at 570 nm on a TriStar2 Multimode Reader LB942 (Berthold Technologies, Bad Wildbad, Germany). Median values of replicate analyses and standard deviations (SD) were calculated using Microsoft Excel. IC_50_ was calculated by non-linear regression analysis using GraphPad Prism 7.0a software.

### 4.7. Migration and Wound-Healing Assays

Migration assays were conducted as previously described and analyzed after 48 h [53]. For wound-healing assays, A673 monolayers of 80% confluence were pre-treated with antibodies for 2 h before a wound was created using a pipette tip. Images were acquired at indicated time points and wound areas were quantified using the MRI Wound Healing Tool plug-in for Image J software [54].

### 4.8. Zebrafish Xenograft Model

The transgenic zebrafish line Tg(kdrl:mCherry), originally received from the D. Stainier’s laboratory [55], provides fluorescently traceable blood vessels. Zebrafish were kept in compliance with local animal welfare regulations and European directives. The study was approved by the local animal welfare committee (DEC) of the University of Leiden (license number 10612, protocol 14227). Zebrafish adults were maintained according to standard protocols in a 10/14-hour dark/light cycle [56]. Larvae were maintained at 28 °C in egg water (60 µg/mL ocean salt in distilled water), containing 0.003% 1-phenyl-2-thiourea to block pigmentation. Experiments were performed on zebrafish larvae before the onset of independent feeding, according to Dutch animal welfare regulation. Experimental procedures and analyses were previously described in detail [57] (also see Document S1: ARRIVE Guidelines Checklist). A673 cells expressing shRNA-silencing RON (Group 1—experimental group) or the non-silencing control (Group 2—control group) together with GFP reporter protein were implanted into the duct of Cuvier of larvae 2 days post fertilization. Four days after implantation, tumor burden was analyzed by automated image analysis of GFP-fluorescent objects per zebrafish area as described [57].

### 4.9. Statistics

Statistical significance of multiple different conditions was calculated using ANOVA with Sidak correction for post-hoc pairwise comparisons. Independent pairwise comparisons were calculated using t-tests, and dependent pairwise comparisons were calculated using t-tests with the Benjamini–Hochberg (FDR) correction. Calculations were performed in Microsoft Excel and GraphPad Prism 7.0a software. Significance is indicated as *p* < 0.05 (*), *p* < 0.01 (**) and *p* < 0.001 (***), while ns indicates a non-significant *p*-value.

## 5. Conclusions

Our study provides a first characterization of the scatter factor receptor RON in sarcomas, specifically Ewing sarcoma. Reflecting on previous findings in carcinomas, our data indicate a function of RON within the micrometastatic cellular capacities of Ewing sarcoma. RON’s benefit as a therapeutic target may therefore lie in slowing of metastatic tumor progression more than tumor proliferation. Our findings further suggest that RON’s net tyrosine kinase activity derives from both ligand-mediated and ligand-independent mechanisms, such as RTK crosstalk and the constitutively active isoforms. For future therapeutic exploitation of RON, a kinase-directed targeting strategy may therefore be more successful than the ligand-dependent antibody approach employed here.

Although this study remains initiatory in many aspects and leaves several open questions, this example of RON highlights that successful therapeutic targeting of RTKs requires an understanding of their diverse mechanisms of kinase activation, relevant receptor isoforms and dynamic interactions in receptor and signal transduction networks, as these may comprise prime therapeutic targets next to resistance mechanisms. Despite an overlap of these principal mechanisms between rare pediatric sarcomas and frequent carcinomas and leukemias, distinct expression patterns and regulations observed in Ewing sarcoma underscore that future advances towards RON and RTK (co-) targeting must consider this tumor-specific background to select the optimal, most effective strategy for each cancer.

## Supplementary Materials

The following are available online at https://www.mdpi.com/2072-6694/12/4/904/s1, Figure S1: RON expression in additional Ewing sarcoma datasets, Figure S2: MET is minimally expressed in Ewing sarcomas and cell lines, Figure S3: RON targeting does not affect cell viability in vitro, Figure S4: Ewing sarcomas express two sfRON variants, Figure S5: Uncropped immunoblots, Document S1: ARRIVE Guidelines Checklist.
