# Supplementary material for: The Receptor Tyrosine Kinase RON and Its Isoforms as Therapeutic Targets in Ewing Sarcoma
Source: Cancers (Basel). 2020 Apr 7;12(4):904. doi: 10.3390/cancers12040904 (PMC7226494; doi:10.3390/cancers12040904)
Supplement: Supplementary file 1 [file cancers-12-00904-s001.zip › Supplementary Figures S1-5.pdf]

Supplementary Figures S1-S5

To: Berning, P; *et al*.. **The receptor tyrosine kinase RON and its isoforms as therapeutic targets in Ewing sarcoma**

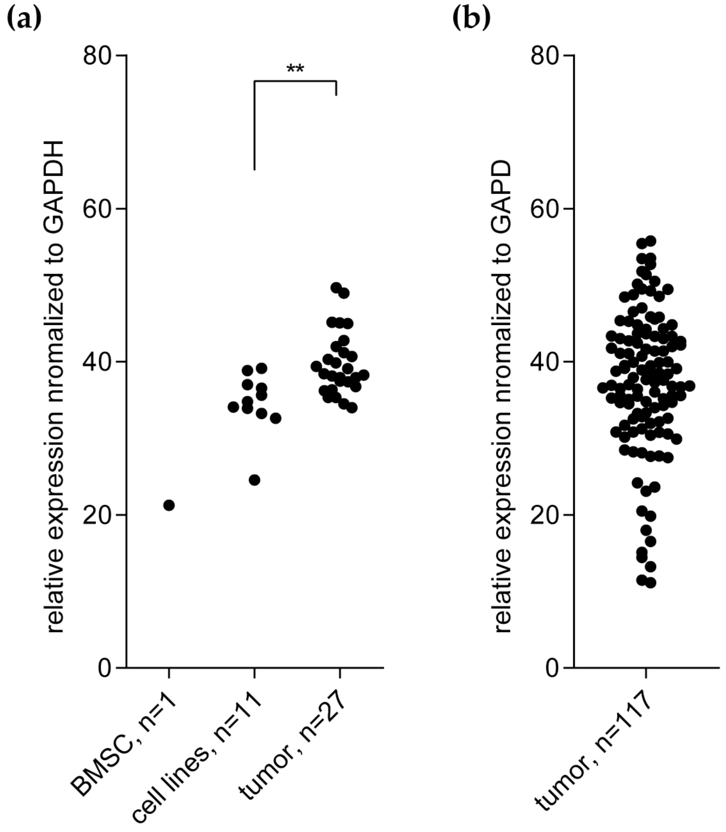

Figure S1. *RON* expression in additional Ewing sarcoma datasets. *RON* and *GAPDH* transcript expression were extracted from two datasets publicly available on the R2: genomics analysis and visualization platform (http://r2.amc.nl). (a) Relative *RON* transcript expression in human primary bone marrow stem cells (BMSC), Ewing sarcoma cell lines and tumors, as determined by analysis of microarray data; dataset as deposited by Tirode et al. [26]; (b) *RON* transcript expression in Ewing sarcomas, as determined by analysis of microarray data; dataset as deposited by Postel-Vinay et al. [27];

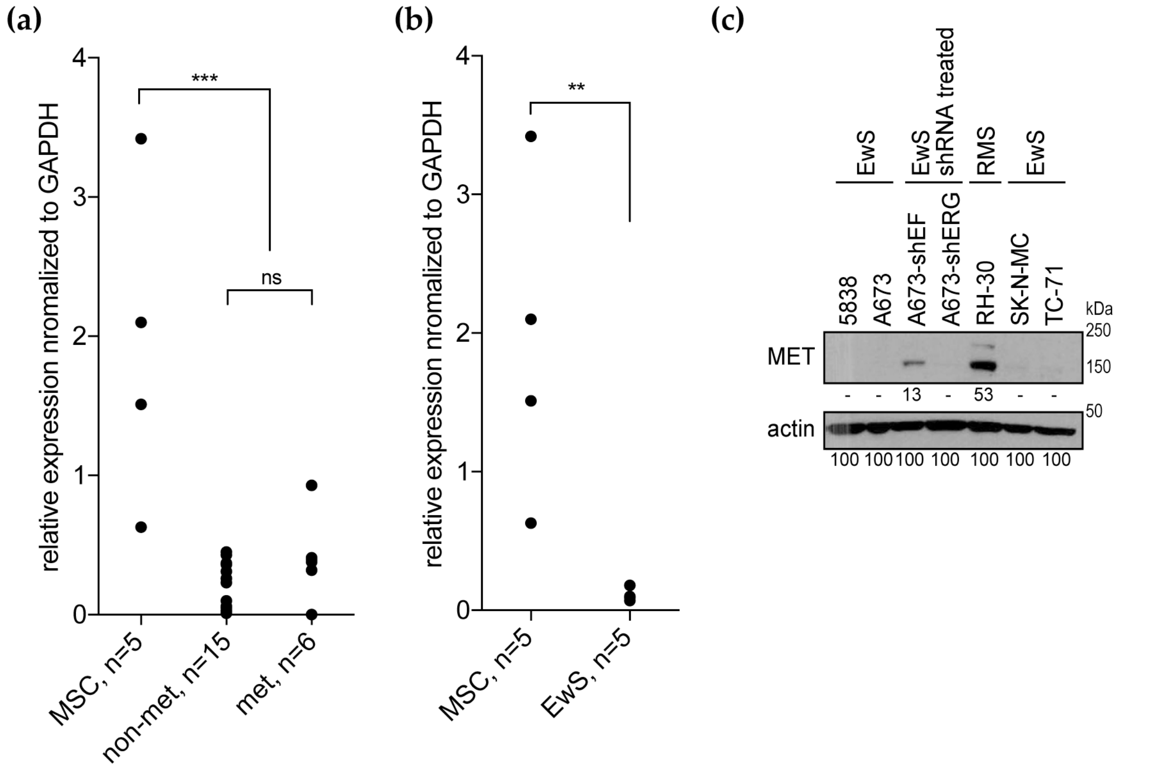

Figure S2. MET is minimally expressed and not active in Ewing sarcomas and cell lines. (a) Relative MET transcript expression in Ewing sarcoma primary tumors from patients with localized (non-met) or metastatic (met) disease in comparison to mesenchymal stem cell cultures (MSC), as determined by qPCR; (b) Respective MET expression in Ewing sarcoma cell lines (EwS) and MSC cultures; (c) MET protein is not expressed in Ewing sarcoma cell lines but is restricted to A673-shEF cells with shRNA-silencing of the Ewing sarcoma-specific EWS-FLI1 oncogene. A673-shERG cells containing shRNA directed against ERG, which is not expressed in A673, serve as control [52]. RH-30 rhabdomyosarcoma cells (RMS) serve as positive control for MET expression and phosphorylation. Cells were grown in standard tissue culture conditions. Arrow indicates double-band of phosphorylated MET. 10% gel; numbers indicate densitometry readings relative to respective actin loading control.

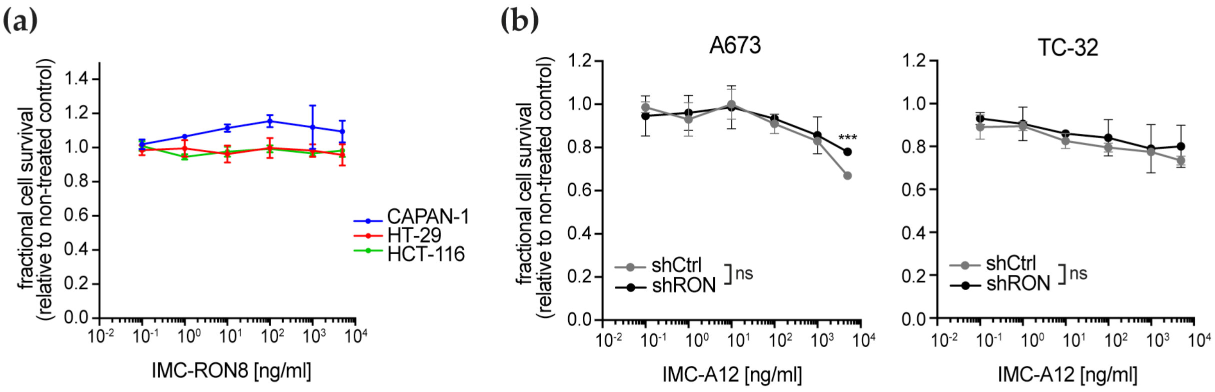

Figure S3. RON targeting does not affect cell viability *in vitro*. (a) The therapeutic anti-RON antibody IMC-RON8 does not significantly reduce monolayer cell viability of HT-29, HCT-116 and CAPAN-1 carcinoma cell lines. Cells were grown in standard conditions and treated as indicated. After 72 h, relative cell viability was measured by MTT assay. Graphs represent mean ± SD of ≥3 independent experiments; (b) Co-targeting of RON and IGF1R by combined shRNA and IMC-A12 approach does not reveal increased effects compared to IMC-A12 alone. 9 days after transduction with shRNA targeting RON (shRON) or non-silencing control (shCtrl), cells were grown, treated and analyzed as in (a). Graphs represent the mean ± SD of ≥3 independent transduction experiments.

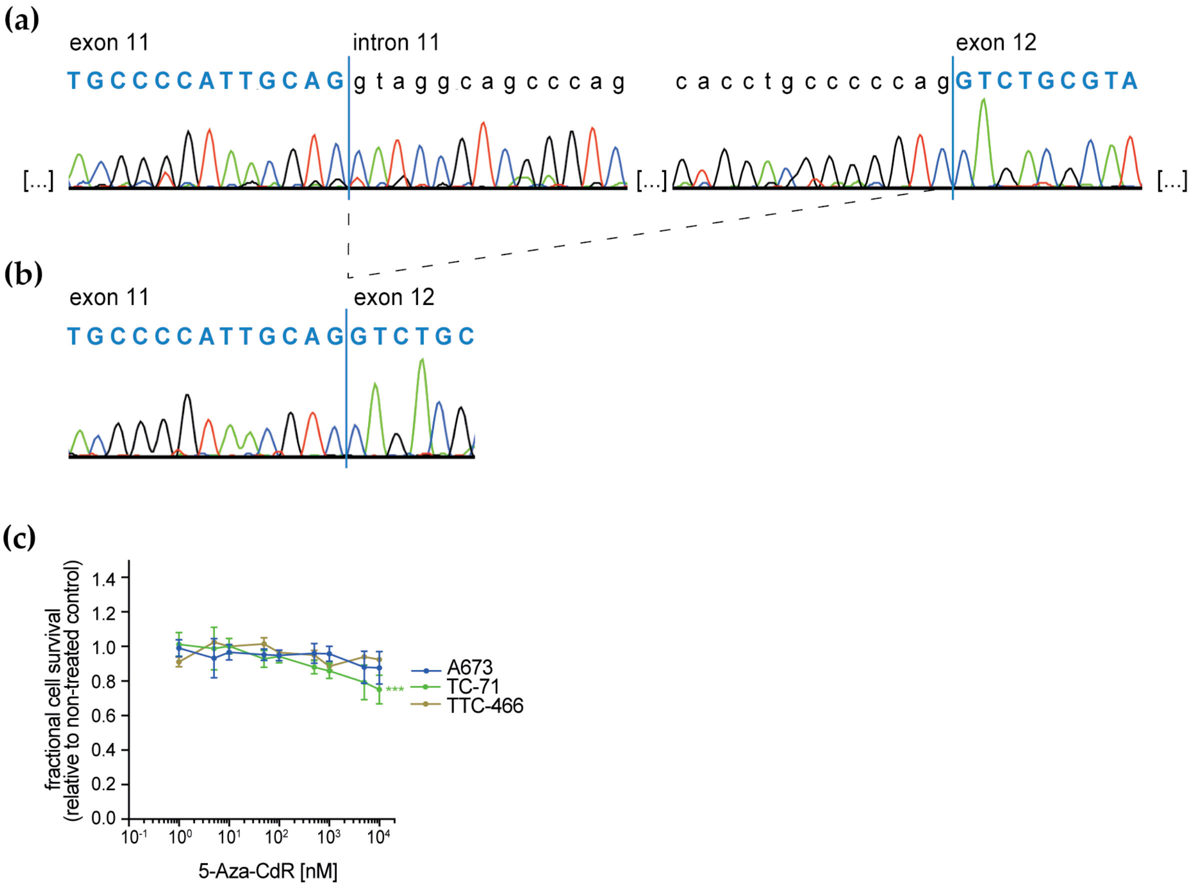

Figure S4. Ewing sarcomas express two sfRON variants. Upper and lower bands of sfRON PCR products from Figure 5b were sequenced, revealing higher-bp bands to contain intron 11 sequences (a), which were spliced in the lower-bp band (b). Representative sequences shown are from sample 9 of Figure 5b; (c) Dose response of Ewing sarcoma cell lines to 5-Aza-CdR treatment. Cells were grown in standard conditions and treated as indicated. After 72 h, relative cell viability was measured by MTT assay. Graphs represent mean ± SD of ≥3 independent experiments.

Figure 1. (c)

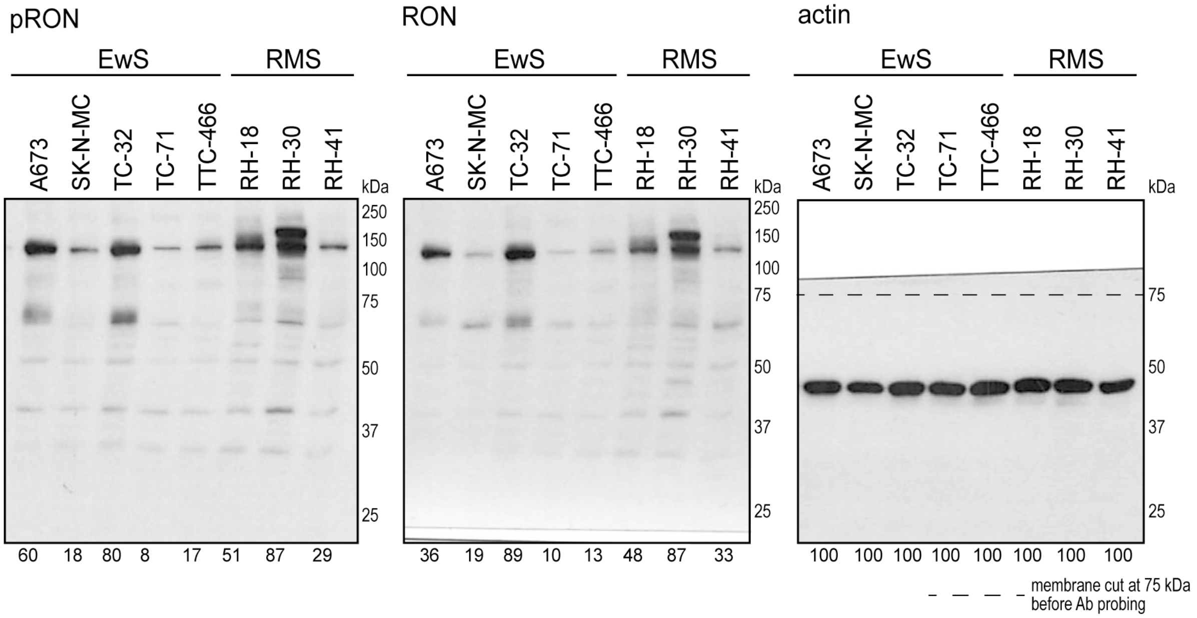

Figure 2. (a)

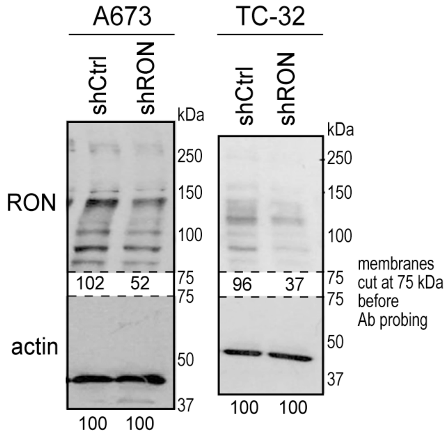

Figure 4. (c)

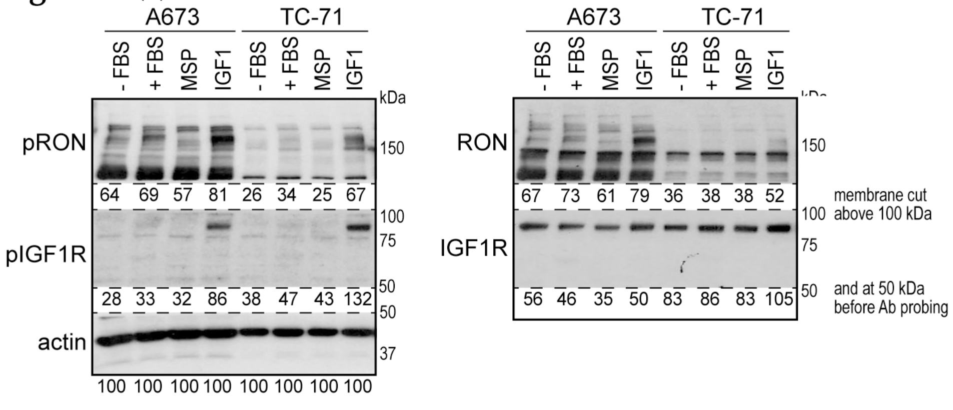

Figure 5.(a)

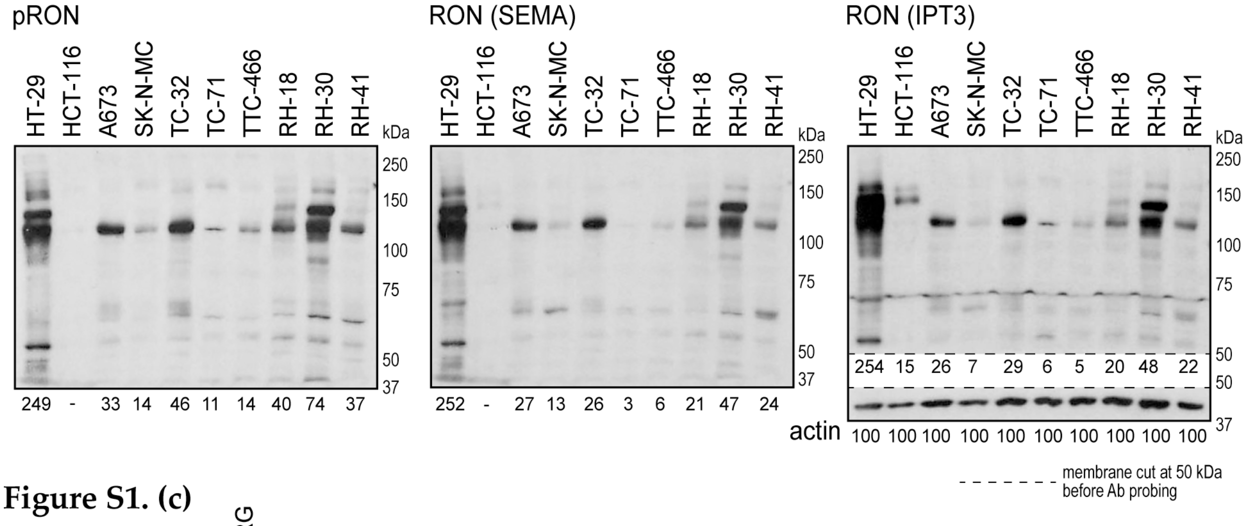

Figure S1. (c)

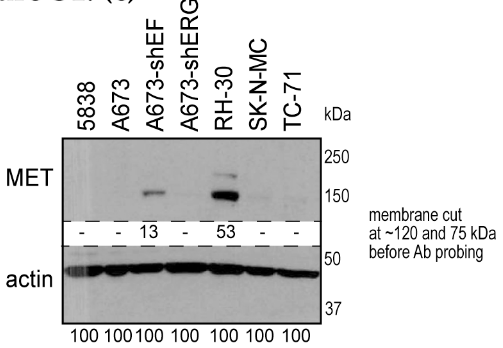

Figure S5. Uncropped immunoblots. In several experiments, nitrocellulose membranes were cut following protein transfer, based on Ponseau-S staining and protein markers, to facilitate comparative analysis of protein levels by parallel probing of primary antibodies. Numbers indicate densitometry readings of full lanes relative to respective actin loading control.
